# Antagonism of the EP2 Receptor Reveals Sex-Specific Protection in a Two-Hit Mouse Model of Alzheimer’s Disease

**DOI:** 10.1021/acschemneuro.5c00780

**Published:** 2026-01-02

**Authors:** Avijit Banik, Radhika Amaradhi, Michael Sau, Varun Rawat, Raymond Dingledine, Thota Ganesh

**Affiliations:** Department of Pharmacology and Chemical Biology, 12239 Emory University School of Medicine, Atlanta, Georgia 30322, United States

**Keywords:** neuroinflammation, behavioral deficits, EP2 antagonism, 5xFAD-SJL, lipopolysaccharide

## Abstract

Neuroinflammation is evident in Alzheimer’s disease (AD) brains, exacerbating the pathology and ensuing cognitive deficits in patients. The prostaglandin-E2 receptor EP2 emerged as a neuroinflammatory target in several neurodegenerative diseases, including AD. Antagonism of EP2 mitigates neuroinflammation and cognitive deficits in status epilepticus and stroke models. Here, we investigated the efficacy of a potent and selective EP2 antagonist TG11–77.HCl on the cognitive behavior and neuroinflammation in a two-hit 5xFAD mouse model of AD. We exposed adult 5xFAD mice on B6SJL genetic background and their nontransgenic littermates to a low dose of lipopolysaccharide and administered TG11–77.HCl or the vehicle in the drinking water for 12 weeks. Mice were subjected to Morris water maze and Y-maze testing during their last week of drug treatment. Blood samples were subjected to complete blood count (CBC) analysis and brain tissues were processed to examine the levels of inflammatory transcripts and glial marker expression (mRNA), followed by the quantification of congophilic amyloid deposition and microglial activation (IBA^+^) in the brain by immunohistochemistry. TG11–77.HCl treatment enhanced the spatial memory performance and ameliorated mRNA expression of proinflammatory mediators, chemokines, and cytokines in the neocortex of 5xFAD males only and attenuated astroglia and microglia activation in both male and female 5xFAD mice and the congophilic amyloid load in 5xFAD males only. CBC analysis revealed no changes in peripheral inflammation, irrespective of sex, on treatment with TG11–77.HCl. This study reveals sex-specific protection of selective EP2 antagonism in a two-hit mouse model of AD and supports a prudent therapeutic strategy against neuroinflammation and associated cognitive impairment in AD.

## Graphical abstract

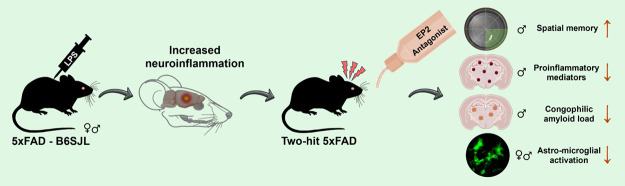

## Introduction

Prostaglandin-E2 (PGE_2_) receptor subtype EP2 has emerged as an important mediator of neuroinflammatory pathology and ensuing cognitive deficits in a variety of acute and chronic brain injury models.^1,2^ For example, in status epilepticus (SE) models using pilocarpine in mice and diisofluoropropyl phosphate in rats, we have shown that a brief exposure of EP2 antagonist attenuates neuroinflammation and ensuing cognitive and memory deficits weeks following SE.^3–5^ Minhas et al. have shown that the inhibition of myeloid EP2 rejuvenates systemic and brain inflammatory states, synaptic plasticity, spatial memory, and cellular bioenergetics.^6^ Furthermore, the inhibition of peripheral myeloid EP2 signaling was sufficient to restore cognition in aged mice.^6^ Recently, we have shown that chronic treatment of two-hit 5xFAD mice on the C57BL6 background with EP2 antagonist TG11–77.HCl beginning from 2–3 months to 5 months of age (i.e., prodromal stage of Alzheimer’s disease (AD)) reduced the inflammatory pathology in only females but not males.^7^ In 5xFAD mice on a C57BL6 congenic background, we found a relatively modest, 2–4-fold, increase in neuroinflammatory gene expression (IL-1β, TNF, IL-6, CCL2, and EP2). Moreover, we found sex-dependent inflammatory gene expression changes in which adult females showed relatively higher gene expression (3–4-fold) compared with males (2–3-fold). The previous study suggested that the EP2 antagonist will have the best efficacy in attenuating neuroinflammation when the neuroinflammatory insult is higher.^7^ Although the amyloid pathology worsened with age in the 5xFAD mice on C57BL6 background; these mice did not show any behavioral and memory deficits as measured up to 11 months of age when tested using Y-maze and elevated plus maze in our laboratory (unpublished). Interestingly, the less commonly used 5xFAD-SJL mouse model, which was originally created,^8^ displayed neuroinflammation in parallel with cerebral amyloid AB_42_ at 2–3 months of age and memory deficit by 4–5 months.^8^ With the anticipation that a mixed genetic background will contribute to more genetic diversity and a pronounced impact on neuroinflammation, we turned our interest to 5xFAD mice with a B6SJL mixed genetic background to validate a proof-of-concept that EP2 antagonism is therapeutically beneficial in AD.

Lipopolysaccharide (LPS) is an activator of the immune system. It binds to the CD14/TLR4/MD2 receptor complex in monocytes, dendritic cells, macrophages, and B-cells, which promote the secretion of prostaglandins, proinflammatory cytokines, nitric oxide, and reactive oxygen species, respectively. It has been shown that LPS induces mild-to-severe systemic inflammation, inflammation in the brain, and behavioral deficits.^9–11^ While a high dose of LPS is associated with serve injury and adverse effects including mortality, a chronic low dose has been shown to induce chronic anemia of inflammation in the blood and brain.^7,12–14^ Therefore, we envisioned that LPS can be given to 5xFAD mice to simulate a two-hit model of AD. We describe here that the EP2 antagonist TG11–77.HCl upon chronic dosing to two-hit 5xFAD-SJL mice suppressed the inflammation and amyloid load in males and suppressed gliosis and spatial memory deficits in male and female 5xFADs.

## Results

### Experimental Paradigm

As shown in Figure 1, this is a prophylactic study in which an EP2 antagonist was delivered from 2 to 5 months of age to determine the impact on neuroinflammatory markers. Both sexes of transgenic (5xFAD) mice and their nontransgenic (nTg) littermates were subjected to once a week intraperitoneal (*ip*) injection of LPS (1 mg/kg). However, TG11–77. HCl (projected nominal dose 100 mg/kg/day) was dissolved in drinking water (at pH 3.5) and provided continuously from 8 to 20 weeks of age (12 weeks total). Control animals received drinking water (adjusted to pH 3.5) as a vehicle. In the previous study, we used 0.5 mg/kg injection of LPS once a week from 12 to 20 weeks of age (8 weeks);^7^ however, only a modest inflammatory signal was found with this dose and duration. Therefore, in the current study, we injected a higher dose of LPS (1 mg/kg per week) and with a longer dosing period (12 weeks) to introduce a more robust effect on neuroinflammation. We had two groups of mice, i.e., Env-hit (environmental hit; LPS in nTg) and two-hit (LPS in 5xFAD) from both sexes, treated with either TG11–77.HCl or the vehicle (Figure 1). The actual dose for the free base of TG11–77.HCl in these mice was calculated to be 53 mg/kg/day based on their weekly body weight and consumption rate of the drinking water along with a further 92.5% recovery of the drug from the stored drug-mixed water after 7 days at room temperature (SI Table S1). The level of peripheral inflammation was reported to vary between sexes;^6^ hence, we included both males and females in this study. There was no adverse effect of the drug on the weight gain of these mice over the period of the study (SI Figure S1).

**Figure 1:**
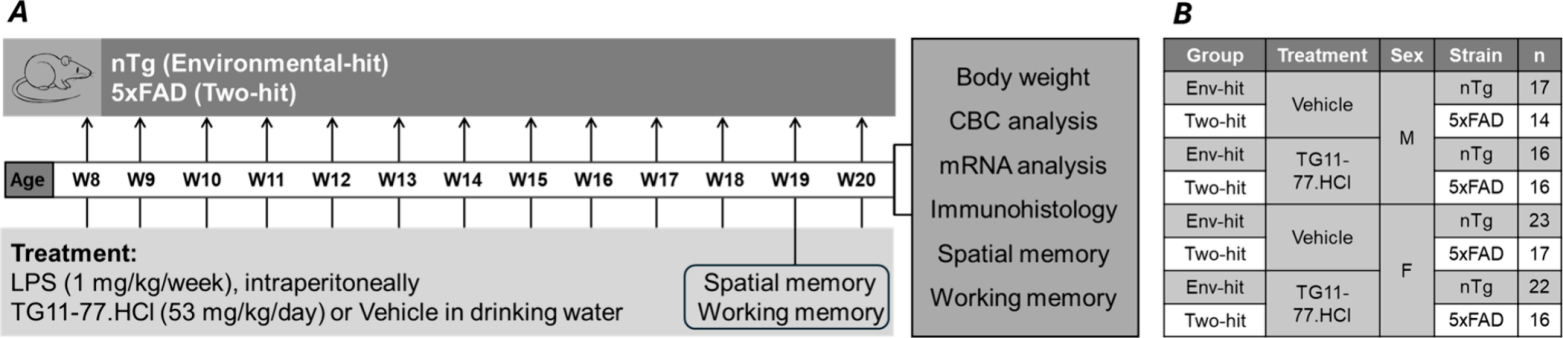
Experimental paradigm used in the study. (A) Both the sexes of nontransgenic (nTg) and 5xFAD mice were subjected to LPS and TG11–77.HCl administration from 8 to 20 weeks of age. Animals were tested for spatial and working memory on the 19th week and perfused with ice-cold PBS 6 h after the last LPS injection (20th week). (B) Table showing all of the group details and animal numbers used. Env-hit = LPS in nTg; two-hit = LPS in 5xFAD.

### TG11–77.HCl Treatment Does Not Alter LPS-Induced Anemia of Inflammation

We have previously shown that the administration of chronic low-dose LPS (0.5 mg/kg/week, for 8 weeks) in 5xFAD or nontransgenic mice created an anemic situation in the complete blood count (CBC) analysis and neuroinflammation (see Figure 2 in ref 7). We anticipated a similar anemic and inflammatory state in the blood and brain of the mice in the current study with an increased dose and duration of LPS (1 mg/kg/week for 12 weeks). Here, we asked if there was any antagonistic effect of TG11–77.HCl on the peripheral anemia of inflammation in both cohorts, Env-hit and two-hit mice (SI Figure S2). The CBC analysis in these mice 6 h after the last LPS dose showed no effect of TG11–77.HCl treatment. The numbers of red blood cells (RBCs), lymphocytes, monocytes, neutrophils and platelets were found to be unchanged in both cohorts (SI Figure S2A,C). Similarly, there was no difference in the levels of hemoglobin (HGB), % hematocrit (HCT), RBC width (RDWc), and platelet distribution width (PDWc) between drug- and vehicle-treated mice (SI Figure S2B,D). Overall, TG11–77.HCl treatment did not alter the LPS-induced anemia of inflammation. We also examined any effects of TG11–77.HCl independently in males and females in these cohorts, but no difference was found (data not shown).

### TG11–77.HCl Changes the mRNA Expression of Proinflammatory Mediators in the Two-Hit Male Neocortex

TG11–77.HCl treatment in two-hit mice attenuated the mRNA expression levels of several proinflammatory mediators, such as COX-2, p47phox, gp97phox, and iNOS, in the neocortex of the male brains (Figure 2). Interestingly, TREM2, known for its anti-inflammatory responses in microglia, was expressed significantly higher in two-hit males and females and was attenuated by the TG11–77.HCl treatment. Although the expression decreases in both sexes, the benefit was much more evident in two-hit male brains (Figure 2F,N). This effect was not found in the neocortex from the Env-hit mice except for iNOS in the males (Figure 2M). The EP2 mRNA level was found to show an increasing trend (statistically nonsignificant) after drug treatment in both male and female Env-hit mice (Figure 2A,G,L,O). However, the EP2 level was marginally decreased in two-hit female and male mice after treatment with TG11–77.HCl (Figure 2A,I). Overall, the anti-inflammatory effect of the EP2 antagonist was evident in two-hit males, projecting a reduced level of the proinflammatory modulators in the brain neocortex, but this effect was statistically nonsignificant (Figure 2P; *p* = 0.053, paired *t* test). We also did not find a statistically significant effect of TG11–77.HCl treatment in two-hit females (Figure 2H; *p* = 0.38, paired *t* test).

**Figure 2:**
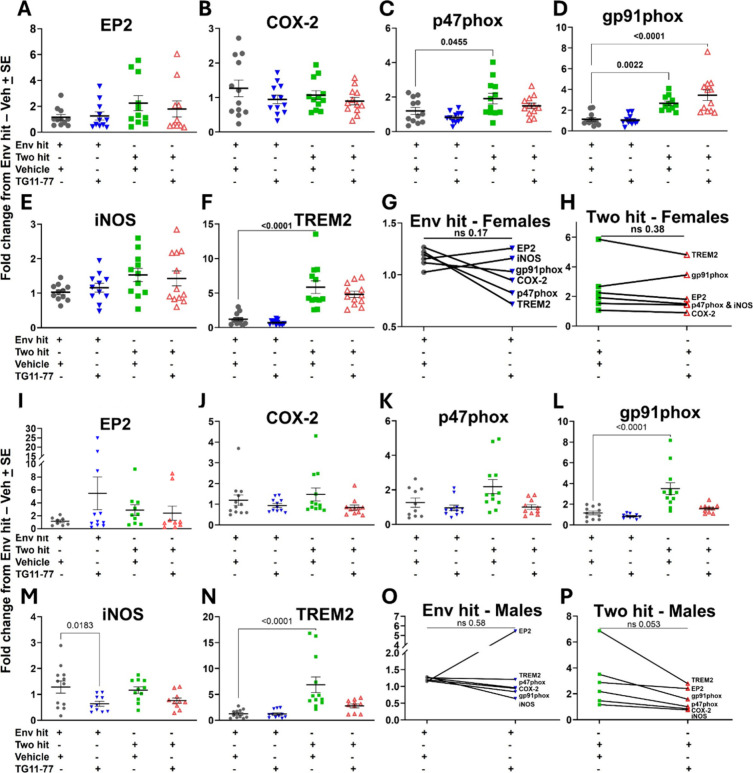
TG11–77.HCl shows a reducing trend in the expression of proinflammatory mediators in the neocortex of two-hit 5xFAD mice. The mRNA fold changes of individual proinflammatory mediators in female (A–F) and male (I–N) Env-hit and two-hit mice are reported. A paired comparison between vehicle and TG11–77.HCl-treated females (G, H) and males (O–P) shows overall trends for all of the proinflammatory mediators. All of the groups were normalized to their Env-hit—vehicle-treated counterparts. For individual end-point analysis, one-way ANOVA with Dunnett’s multiple comparisons test was applied (A–F, I–N). For group analysis between different hits, a paired *t-*test was applied (G, H, O, P). Data are mean ± SEM; ns = nonsignificant.

### TG11–77.HCl Attenuates the mRNA Expression of Inflammatory Chemokines and Cytokines in the Two-Hit Male Neocortex

We found that LPS and the presence of the 5 mutations (genetic aberration) both induce higher gene expression of selected chemokines and cytokines in the two-hit male neocortex compared to females (Figure 3). IL-1β, IL-4, and IL-18 expressions were found to be similar for both the Env-hit and the two-hit mice (in both sexes) (Figure 3A–D,K–N), whereas IL-6 expression was found to be significantly higher in both male and female two-hit cohorts from their Env-hit counterparts. TNFα and CCL2 expressions were found to be higher by 2–3-fold in the two-hit male compared to their Env-hit cohorts but remained nonsignificant (Figure 3L,O,P). Compared to other chemokines, there was a greater increase in the CCL3 (10–40 folds) and CCL4 (15–30 folds) expression in both male and female two-hit mice (Figure 3G,H,Q,R), suggesting a considerably elevated degree of chemokine-mediated neuroinflammation in the two-hit models. Furthermore, the TG11–77.HCl treatment showed an overall attenuating effect on these chemokines and cytokines in the two-hit males (Figure 3T; *p* < 0.0001, paired *t* test), but it was not as effective in the two-hit females (Figure 3J; *p* = 0.12, paired *t* test). Interestingly, the TG11–77.HCl treatment could also reduce multiple chemokine and cytokine levels in the Env-hit mice (both males and females), but statistical significance was not attained (Figure 3I,S).

**Figure 3:**
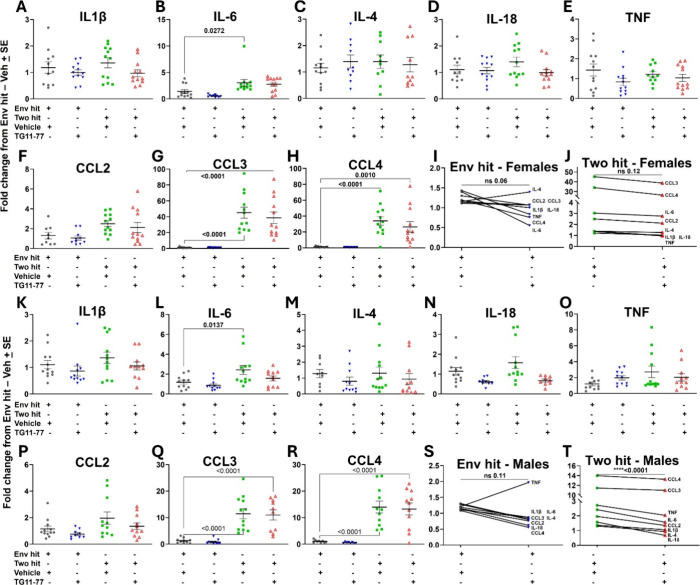
TG11–77.HCl attenuates expression of inflammatory chemokines and cytokines in the neocortex of two-hit 5xFAD males. The mRNA fold changes of individual inflammatory cytokines and chemokines in female (A–H) and male (K–R) Env-hit and two-hit mice are measured. Paired comparison between vehicle- and TG11–77.HCl-treated females (I, J) and males (S, T) shows overall trends for all of the inflammatory chemokines and cytokines. All of the groups were normalized to their Env-hit vehicle-treated counterparts. For individual end-point analysis, one-way ANOVA with Dunnett’s multiple comparisons test was applied (A–H, K–R). For group analysis between different hits, the paired *t-*test was applied (I, J, S, T). Data are mean ± SEM ns = nonsignificant.

### TG11–77.HCl Attenuates the mRNA Expression of Activated Astrocyte and Microglia Markers in Two-Hit Male and Female Neocortex

Similarly, we also measured the gene expression of astrocyte and microglia markers, including GFAP, CD68, Iba1, CD11b, and S100B in the Env-hit and the two-hit mice upon TG11–77.HCl treatment (Figure 4). In both male and female two-hit 5xFAD mice, the mRNA levels of these markers were increased by 2–5-fold compared to the Env-hit nTg mice (Figure 4A–E,H–L). The mRNA levels in the neocortex were found to be significantly lower upon TG11–77.HCl treatment in the two-hit 5xFAD females (Figure 4G; *p* = 0.004; paired *t-*test) and males (Figure 4N; *p* = 0.012; paired *t-*test). TG11–77.HCl treatment also reduced these mRNA markers in the Envi-hit mice, but a statistical significance was not attained in both sexes (Figure 4F,M).

**Figure 4:**
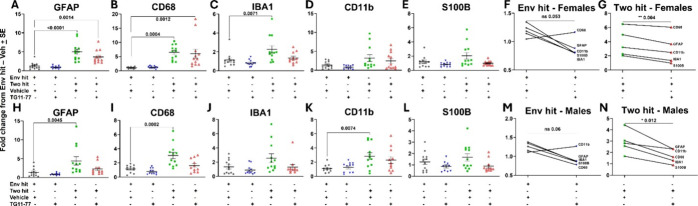
Astroglial and microglial inhibition by EP2 antagonist treatment in two-hit 5xFAD males. (A–E) Effect of TG11–77.HCl treatment on Env-hit and two-hit females. (H–L) Effect of TG11–77.HCl treatment on Env-hit and two-hit males. The fold changes in all of the groups were normalized to their respective nTg (Env-hit/Veh groups) mice. Pairwise effect of TG11–77.HCl treatment in Env-hit and two-hit females (F, G) and males (M, N). For individual end-point analysis, one-way ANOVA with Dunnett’s multiple comparisons test was applied (A–E, H–L). For group analysis between different hits, the paired *t-*test was applied (F, G, M, N). Data are mean ± SEM; ns = nonsignificant.

### TG11–77.HCl Attenuates Microgliosis in Different Brain Regions of Two-Hit Mice

To further validate our gene expression findings, we also examined the brain sections of two-hit mice to determine the impact of TG11–77.HCl treatment on microglia activation by Iba1 immunohistochemistry. We found that the expression of the activated microglial specific protein Iba1 was significantly attenuated by TG11–77.HCl treatment in different regions of the brain in both male and female two-hit mice (Figure 5A,B). All brain regions examined including the amygdala, thalamus, hypothalamus, cerebral cortex, entorhinal cortex, piriform cortex, and hippocampus (CA1, CA2, CA3) showed a decreased expression of Iba1 after the drug treatment. The TG11–77.HCl treatment led to a small but significant overall reduction in the expression of Iba1 by 15% in two-hit males (*p* < 0.01) and 24% in females (*P* < 0.001) (Figure 5A,B). Histological images revealed lower microglial loads in the cortex, thalamus, and amygdala upon TG11–77.HCl treatment (Figure 5C).

**Figure 5:**
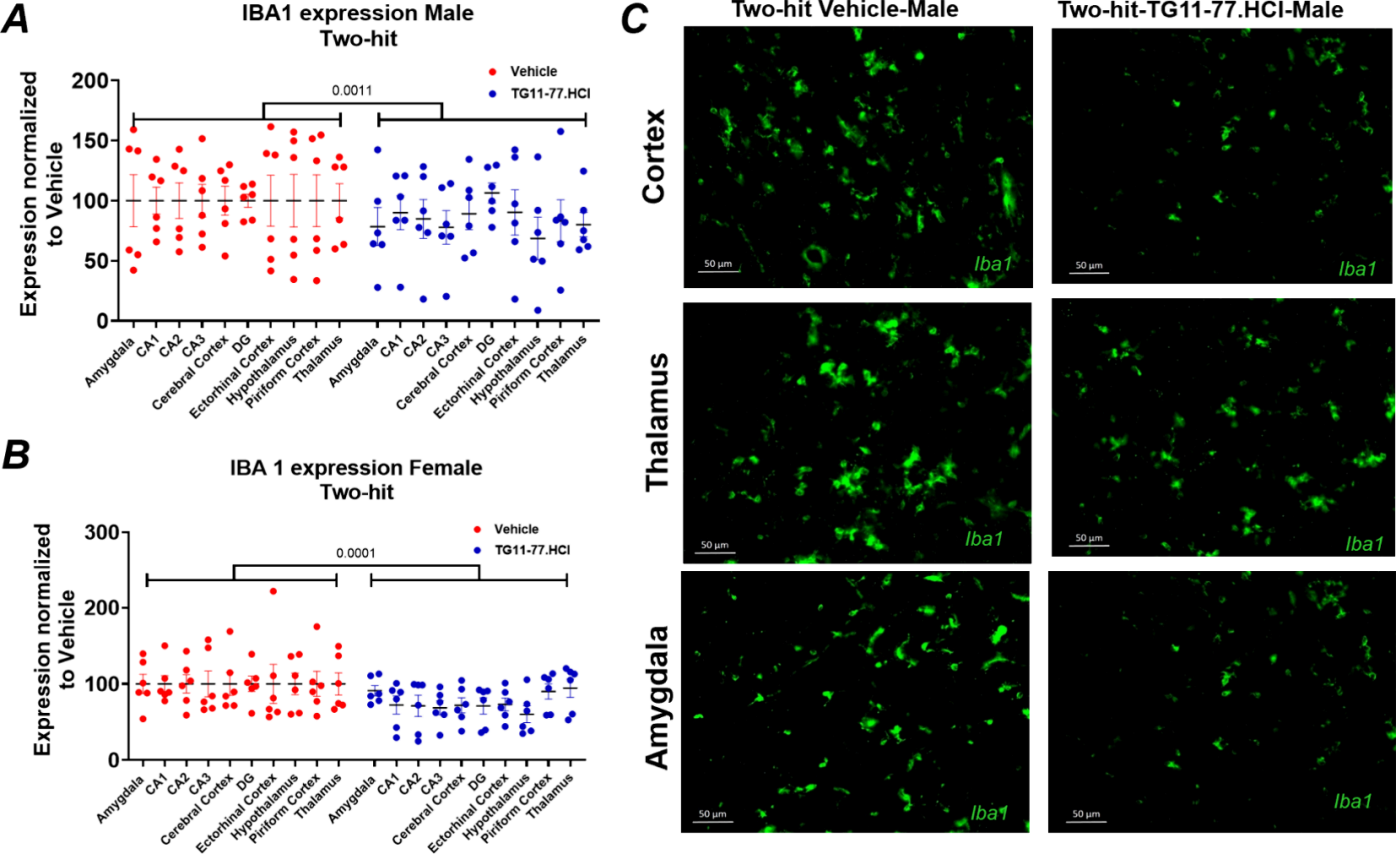
Effect of TG11–77.HCl on the Iba1 immunohistochemical expression in two-hit brains. Activated microglia protein, Iba1, expression was measured in different brain regions of two-hit 5xFAD: (A) males and (B) females treated either with vehicle (drinking water pH 3.5) or with TG11–77.HCl. (C) Representative microscopic images showing the load of activated microglia in the cortex, thalamus, and amygdala from two-hit males compared between vehicle and TG11–77.HCl treatment. The % area covered by Iba1 staining was calculated from the microscopic images at different brain regions using a standard protocol in ImageJ. The % area for each individual region was normalized to the area in the vehicle-treated mice considered at 100%. The paired *t-*test was applied for between-group comparisons. Scale bar: 50 mm. Data are mean ± SEM.

### EP2 Antagonist TG11–77.HCl Treatment Attenuated Amyloid Pathology in Two-Hit Males

To evaluate the effect of TG11–77.HCl treatment on the amyloid pathology, we performed Congo red staining in brain sections from the vehicle- and drug-treated two-hit mice. The cerebral cortex, entorhinal cortex, piriform cortex, amygdala, hippocampus, and thalamus in coronal brain sections were stained and visualized for Congo-red-positive plaques. We measured and compared Congo red staining between the vehicle and drug treatment. TG11–77.HCl treatment reduced the amyloid deposition in different brain regions of two-hit males. The number of amyloid plaques, their size, and the area covered by them in these mice were found to be significantly attenuated by TG11–77.HCl treatment (Figure 6D–F). However, we did not find this attenuating effect in the two-hit females where only the number of plaques showed a downward trend after drug treatment, but the differences from vehicle treatment were not found to be statistically significant (Figure 6A–C). Fluorescent images revealed that the number and size of the plaques were reduced in two-hit males by TG11–77.HCl treatment in different brain regions (Figure 6G–L).

**Figure 6:**
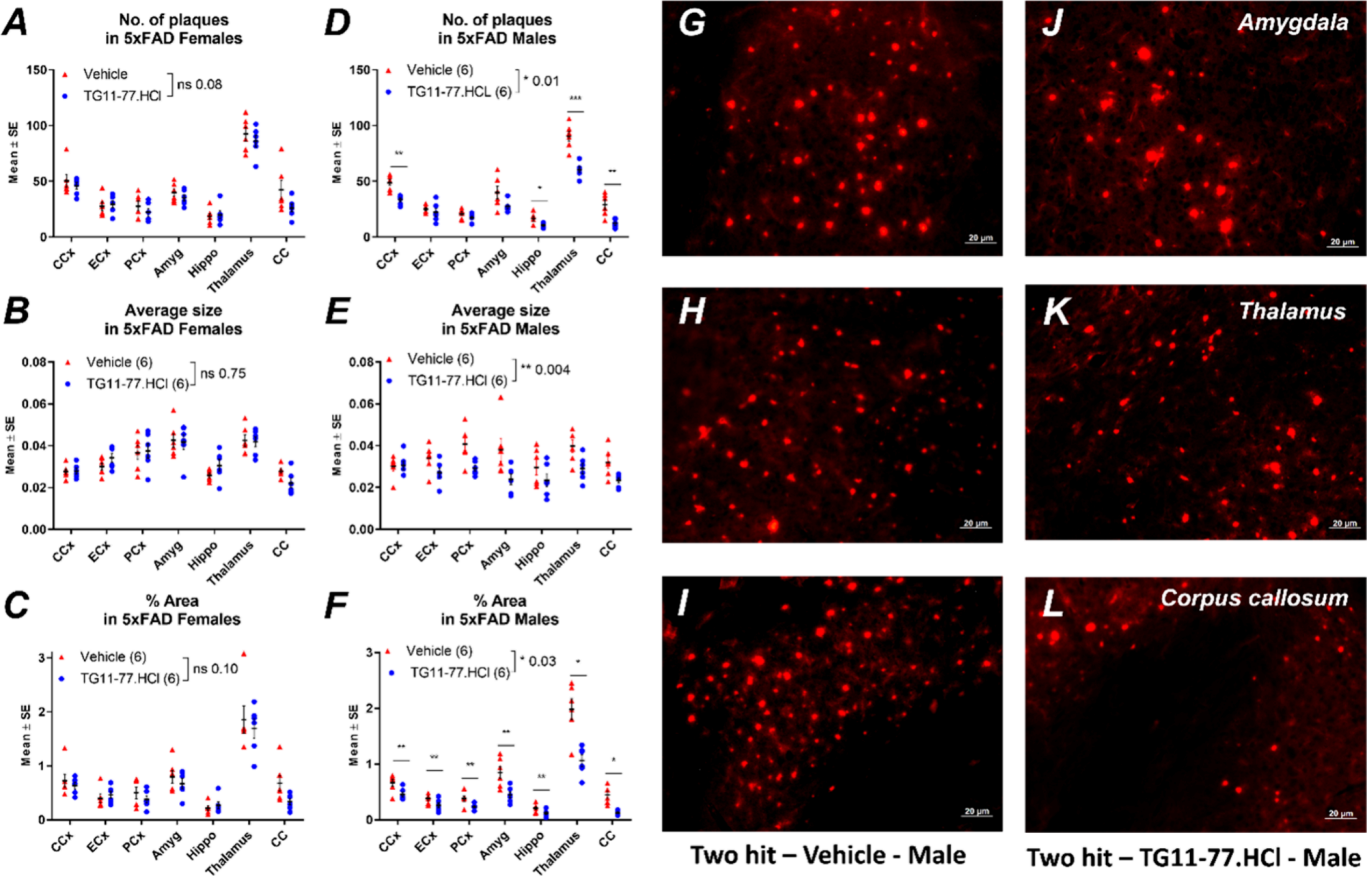
Amyloid deposition in two-hit males and females after TG11–77.HCl treatment. In two-hit female brains, there was nonsignificant change in (A) the number of amyloid plaques, (B) their average size, and (C) the % area covered by them. In two-hit males, (D) the number, (E) size and, (F) % area of the plaques were significantly attenuated by TG11–77.HCl treatment. (G–L) Congo-red-stained microscopic images of Two-hit males show amyloid deposition from different regions of the brain. The between-groups paired *t*-test was applied, and for multiple comparisons, the multiple unpaired *t*-test with FDR (5%) was applied. Data are mean ± SEM; *CCx,* cerebral cortex; *ECx*, entorhinal cortex; *PCx,* piriform cortex; *Hippo,* hippocampus; and *CC,* corpus callosum. ns = nonsignificant.

### TG11–77.HCl Improves Spatial Memory Retention by MWM Testing in Two-Hit 5xFAD Mice

To further evaluate the effect of LPS and the 5 mutations (two hits) as well as the effect of TG11–77.HCl treatment on cognition, we tested the spatial memory performance in the mice using the Morris Water Maze (MWM). All mice were tested in the MWM a week before their final LPS injection. A 5-day protocol was used in which days 1–4 comprised 12 acquisition trials and day 5 had a single probe trial to assess retention memory (Figure 7). Mice from all four groups (Env-hit—vehicle, Env-hit—TG11–77.HCl, two-hit—vehicle, two-hit—TG11–77.HCl) were tested for their memory acquisition and retention index. In acquisition trials, all groups showed a gradual decrease in their escape latency time (ELT) as an index of learning from trials 1 to 12. We did not find any significant difference in the index of learning between the groups. However, within the groups, when we compared the rate of learning, in two-hit females treated with TG11–77.HCl, the ELT (time to reach the hidden platform) showed a significant reduction as early as trial 5 compared to trial 1 (p** = 0.003). To reach a significant reduction in ELT in the vehicle-treated two-hit females, they needed until trial 11 (p** = 0.007) (Figure 7A). Similarly, in two-hit males treated with TG11–77.HCl, ELT was significantly reduced at trial 9 compared to trial 1 (p*** = 0.0003), which was found only at trial 12 (p* = 0.015) for vehicle-treated two-hit males (Figure 7B). During probe trials on day 5, the index of memory retrieval (time spent in the target quadrant, Q3) showed a narrow but significant increase in TG11–77.HCl-treated two-hit females as they spent 22.0 s (p** = 0.003) on average out of 60 s in the Q3 compared to 20.8 s (p* = 0.02) spent by the vehicle-treated two-hit females (Figure 7C). This difference was much larger in two-hit males, as the TG11–77.HCl-treated mice spent in average 27.5 s in Q3 (significantly higher than the average of rest of the other quadrants; p**** < 0.0001), and the vehicle-treated mice spent 22.9 s in Q3 (p** = 0.004) (Figure 7D). This refined performance by the TG11–77.HCl-treated two-hit males in retrieval trial on day 5 was further shown in the heat maps by the yellow patch around the hidden platform zone in the target quadrant (Q3), revealing the mice spending a greater amount of time around the hidden platform as an index of memory retention (Figure 7L), which was not seen in vehicle-treated two-hit males (Figure 7K).

**Figure 7:**
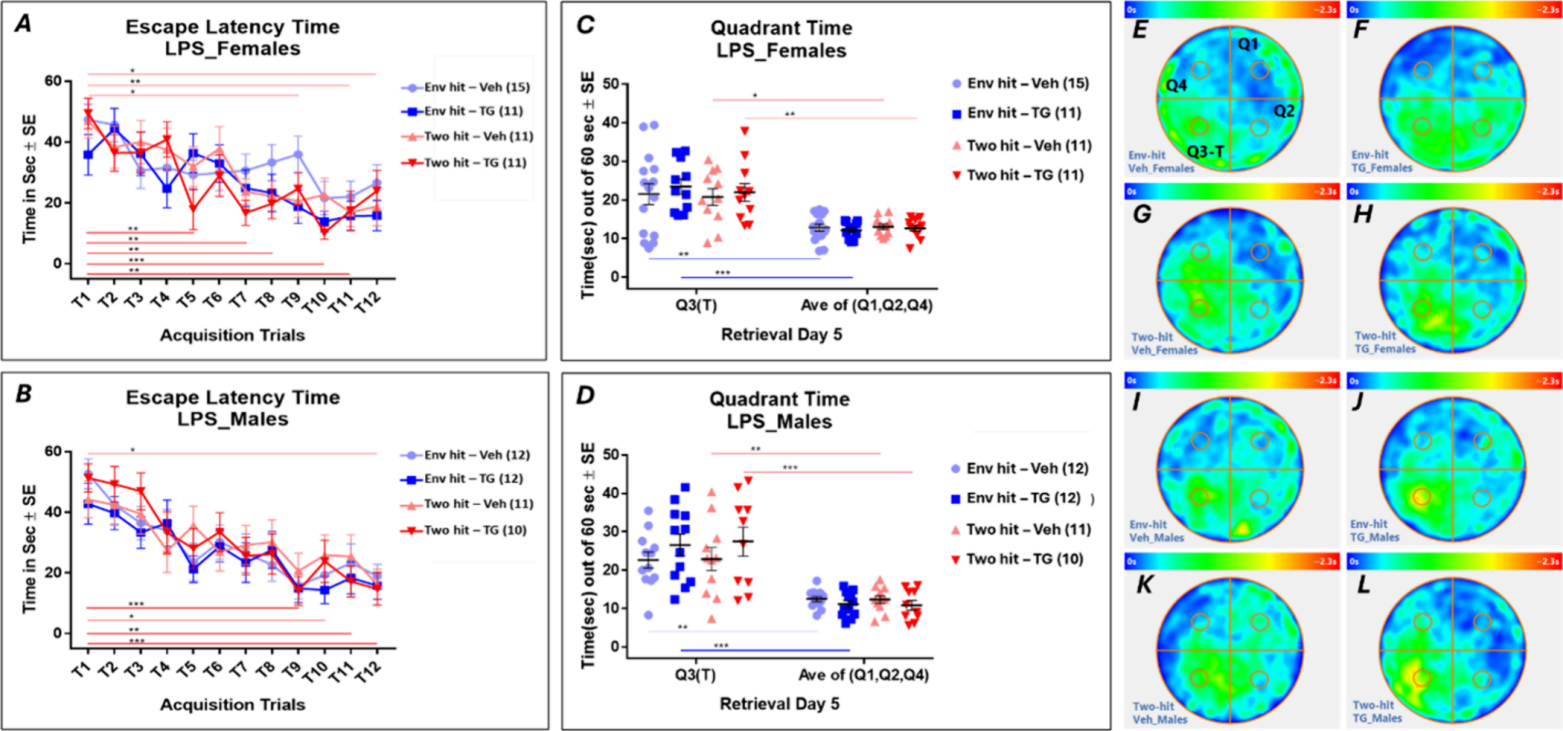
Spatial memory performance in the MWM by TG11–77.HCl- or vehicle-treated mice. On day 1–4, the ELT was measured as an index of memory acquisition, where the average time was recorded for each group to reach the hidden platform kept at target quadrant, Q3. On day 5, the time spent in the target quadrant (probe trial) was measured as an index of memory retrieval. (A) ELT for two-hit or Env-hit females treated either with vehicle of drug. (B) The ELT for two-hit or Env-hit males treated either with vehicle of drug. (C) Quadrant time in two-hit or Env-hit females treated either with vehicle of drug. (D) Quadrant time in two-hit or Env-hit males treated either with vehicle of drug. (E–L) Heat maps of probe trials from different groups showing swimming preferences of these mice at different quadrants of the MWM pool. Quadrants are marked as Q1, Q2, Q3-T, and Q4, whereas Q3-T was the target quadrant having the hidden platform during acquisition trials 1–12 for days 1–4. During retrieval (probe) trials on day 5, the hidden platform was removed from the pool. In heat maps, the time spent in the pool is represented by sea green → green → yellow with increasing time spent in the quadrants. Two-way repeated measures ANOVA with Tukey’s multiple comparison test was applied for acquisition trials. Two-way ANOVA with Sidak’s multiple comparison test was applied for retrieval trials. *P*-values were set to be significant at * ≤ 0.05, ** ≤ 0.001, and *** ≤ 0.0001. Data are mean ± SEM.

We also examined the effect of TG11–77.HCl on the working memory performance of these mice. We measured the percent alternation in the Y-maze, where the preference of the mice toward alternating arms of the Y-maze was recorded. A normally performing mouse does not prefer to enter the most recently visited arm in this test, demonstrating their exploratory behavior. It is only when their spatial memory was impaired that they repeated the arm entries consecutively. Interestingly, the Y-maze performance from all of the groups in our study showed that the working memory of the mice, irrespective of environmental or genetic hits, and vehicle or drug treatments, remained unaltered (Figure S3). The percent alternation in the three arms was found to be similar between vehicle- and TG11–77.HCl-treated Env-hit and two-hit mice (Figure S3A,B). The heat maps from their Y-maze performance showed indistinguishable arm preferences between different groups of mice irrespective of their gender (Figure S3I–L).

## Discussion

AD is a multifaceted chronic neurodegenerative disease with multiple risk factors including but not limited to genetics (familial), epigenetics, environment (exposure to metals, pollution), sleep, diet, severe head injuries (traumatic injuries), infections (bacteria, viruses), and aging-related other illnesses.^15^ While amyloid and tau pathologies were identified as pathological markers in AD brain that contribute to cognitive dementia, inflammation in the brain (aka, neuroinflammation) was viewed as a bystander in the developing AD brain for several decades. Neuroinflammation has recently emerged as a pathological marker that causes secondary damage exacerbating disease progression and ensuing cognitive and memory deficits.^16^ Animal models that portray a multifaceted feature of AD can contribute to investigating novel therapeutics, rather than the models that only have a genetic manipulation to represent <2% familial patient population. The 5xFAD mouse model appeared as an excellent model for displaying amyloid load (AB_42_) and neuroinflammatory sequelae early in age (at the prodromal stage).^8^ However, the eventual cognitive defects in these mice depend on the sex, age, and genetic background on which they are created/crossed.^17,18^ To our surprise, our prior work on these mice (5xFAD) on C57BL6 background did not show robust inflammatory signals at 5 months of age (measured through the induction of neuroinflammatory cytokines and chemokines and gliosis) in both sexes. Female 5XFADs trended slightly higher in displaying these neuroinflammatory markers compared with males, although their relative expressions compared with the age- and sex-matched wild-type controls were modest (<3-fold in males, < 4-fold in females).^19^ Therefore, we subjected the 5xFAD-C57BL6 mice to a chronic low dose of LPS (0.5 mg/kg, per week, 8 weeks) from 3 months until 5 months, at the prodromal stage of AD, to have a two-hit model.^7^ While this two-hit model displayed anticipated anemia of inflammation in the blood, this model showed similar neuroinflammatory pathology as a genetic single-hit model (5xFAD-C57BL6).^7^ In the current study, we used 5xFAD mice on a B6SJL mixed background and injected LPS dose (1 mg/kg per week, 12 weeks), starting at 2 months until 5 months of age. We decided to use the mixed genetic background (B6SJL) for our AD model in this study, as they are reported to express more robust AD pathology, compared to the congenic 5xFAD model on C57BL/6 background (https://www.jax.org/strain/006554). Therefore, we anticipated that the 5xFAD on B6SJL coupled with the increased LPS concentration and dosing window (1 mg/kg/week, 12 weeks) would give us a strong neuroinflammatory pathology, which would translate to poorer performance in spatial memory and other behavioral tests that we intend to test with small molecule therapeutics.

Our overarching goal is to determine the anti-inflammatory efficacy and beneficial effects on memory deficits in an AD model with a small-molecule EP2 antagonist. In the previous study, on the chronic dosing of TG11–77.HCl through drinking water (starting from 2 months until 5 months) in two-hit 5xFAD-C57BL6 mice, an anti-inflammatory effect of the antagonist TG11–77.HCl was found in females, but not males.^7^ When the genetic background has changed to mixed B6SJL, the antagonist treatment (at 2 months until 5 months) resulted in the attenuation of neuroinflammation and congophilic amyloid load in males and gliosis in males and females, and it enhanced the spatial memory performance in males and females, giving a mixed indication that the efficacy is more pronounced in males than in females. We previously ruled out the possibility of differences in TG11–77 exposures in both sexes based on pharmacokinetic features (in the plasma and the brain).^7^ Therefore, it is possible that the EP2 antagonist treatment may provide gender-specific efficacy and also depend on the genetic background. Nonetheless, additional experiments to find the optimized treatment regimen that will work for both sexes on multiple backgrounds are needed to validate the notion that the gender-specific efficacy of antagonists is real.

A vast majority (>95%) of late-onset Alzheimer’s disease (LOAD) patients develop cognitive dementia that was not due to mutations in familial genes (amyloid precursor protein (APP) or presenilin-1 or −2 (PS1 or PS2)), hinting at factors other than genetics exacerbating or promoting the disease. Infections by viruses, bacteria, and parasites have been investigated as possible triggers for neuropathology for decades. Studies report that AD (and Parkinson’s disease) patients may have been exposed to bacterial infections, and vaccination against common bacterial infections is associated with a decreased risk of AD.^20^ Infections by bacteria or viruses create a peripheral immune response that initiates neuroinflammation. Along these lines, a multiple-hit or two-hit hypothesis has been postulated for the investigation of therapeutic strategies for several neurodegenerative diseases including AD.^21^ The two-hit model we created and used in the past (5xFAD-C57BL6 with 0.5 mg/kg/week, 8 weeks) and now (5xFAD-SJL with 1 mg/kg/week, 12 weeks) produced chronic anemia of inflammation in the blood (SI Figure S2 and ref 7). Interestingly, we have not found the impact of the EP2 antagonist TG11–77 treatment on key blood biomarkers such as RBCs, lymphocytes, and monocytes; RBC or platelet distribution either in the single-hit (Env-hit) or two-hit model (SI Figure S2) suggest that TG11–77 efficacies arise from its actions in the CNS, but not in the periphery, similar to our previous findings.^7^

The effect of EP2 antagonist TG11–77 treatment in reducing microgliosis is consistent in both 5xFAD males and females in this mixed (B6SJL) background. This finding is also consistent with mRNA levels (Figure 4) and IBA+ microglia protein by histology (Figure 5). Several studies have shown that microglia activation is triggered at the early stages of AD.^22^ Microglia, resident immune cells in the brain, are initially activated to counter invading pathogens or unusual debris (e.g., pathogens or amyloid plaques) in the brain with their regular housekeeping and homeostatic function. However, during the progression of AD with increasing amyloid load and neuroinflammatory reaction, microglia are converted from anti-inflammatory to proinflammatory nature, losing effectiveness in performing phagocytosis and other homeostatic functions and releasing proinflammatory cytokines and chemokines, leading to neuronal damage and neurodegeneration.^23,24^ It will be crucial to maintain “the good” (anti-inflammatory and pro-phagocytic) microglia during the advancement of AD by suppressing early or sustained activation. EP2 expressed in microglia not only exacerbate the proinflammatory nature of microglia but also promote microglia-mediated sequestration of glucose into glycogen, reducing the glucose flux and mitochondrial respiration.^6^ Since aging cells heavily depend on glucose as a fuel source, the role of EP2 is detrimental in multiple ways during the advancement of aging and AD.^6,25^ Gratifyingly, we found that EP2 antagonism with chronic dosing of TG11–77 started at the prodromal stage (2 months, until 5 months), decreased the activation of glial markers (IBA1, CD68 for microglia; and GFAP, S100B for astrocytes) measured through mRNAs (Figure 4) and also IBA+ microglia protein by histology (Figure 5). These findings complement the recent results by Minhas et al., in which the administration of a brain-permeable EP2 antagonist **C52** for 1 month restored the youthfulness of microglia by controlling pro- and anti-inflammatory factors in the hippocampus, reducing glycogen synthesis from glucose, and reversing the age-associated spatial memory deficits.^6^ Our results also expand on studies that showed that EP2 antagonist **C52** treatment enhances the amyloid-β phagocytosis by macrophages in in vitro cultures,^26^ and conditional deletion of EP2 from microglia enhanced the phagocytosis of Aβ peptides^25,27^ and prevented the memory deficits measured through novel object recognition tests.^25^ These studies prompt the advancement of an EP2 antagonist for clinical investigation in the treatment of AD.

In this study, EP2 antagonist TG11–77 treatment also reduced the congophilic amyloid plaque load (number of plaques, average size, and % area covered) in subregions of the cortex, hippocampus, and amygdala. However, this result was found only in 5xFAD two-hit males but not in 5xFAD two-hit females. These results are in contrast to our previous results, in which the EP2 antagonist TG11–77 showed an increase in the % area covered by congophilic amyloid plaques but no effect on the number of plaques and average size; moreover, we only found this in females.^7^ Based on our hypothesis, the attenuation of gliosis should be coupled with the enhanced clearance of amyloid plaques (i.e., reduced amyloid-β plaque number and size), which is found in this study in males but not in females. However, in our previous study, we also found attenuation of microgliosis in two-hit females, but this was not translated to a reduction in the amyloid load in females. It is difficult to offer any logical explanation for these anomalous results at this time; therefore, these findings must be validated in an independent model of AD, in which neuroinflammation, gliosis, and amyloid load are exceptionally higher than those found at the prodromal stage we have tested thus far.

TREM2 is primarily expressed in microglial cells and is known to regulate phagocytosis and immune responses in the brain. In AD brains, TREM2 expression is reported to be decreased in multiple studies, whereas specific variants of TREM2 are flagged as the associated risk factors in AD pathogenesis.^28^ Interestingly, in our study, TREM2 expression was found to be increased in two-hit mice compared with Env-hit mice (Figure 2F,N). We also determined TREM2 expression in our single-hit cohorts and the trend was similar (data not shown here). Furthermore, TREM2 expression was found to be downregulated by TG11–77.HCl treatment. This suggests an involvement of TREM2 variants in these mice, supporting the associated neuroinflammation in the brain, which is attenuated by TG11–77.HCl treatment.

Overall, our mRNA data on proinflammatory mediators, cytokines and chemokines, and astroglial and microglial markers indicates that the TG11–77.HCl, an EP2 antagonist, could exert a potent anti-inflammatory effect on 5xFAD two-hit males on mixed SJL genetic background compared to females on the same background. LPS largely induced additional neuroinflammation in the AD brains and therefore allowed the drug to act on the two-hit brains. This was confirmed in our previous study that when the 5xFAD mice without any Env-hit (no LPS) were treated with TG11–77.HCl, the EP2 antagonist could not exert any anti-inflammatory effects in their brain.^7^ Hence, we chose not to include this cohort in the present study.

Although no difference in the spatial memory performance was found between mice treated with the drug or the vehicle, an index of faster learning acquisition and memory retrieval was demonstrated by the TG11–77.HCl-treated two-hit mice (Figure 7). The findings from Y-maze indicated that all groups performed at near-random levels, i.e., no demonstration of working memory in any group (Figure S3). This randomness could be due to the LPS itself reducing the working memory in these mice as earlier data from our lab observed similar outcomes, showing impaired memory acquisition in a novel object recognition test following systemic inflammation by LPS.^9^ This finding emphasizes not a robust but a positive impact of anti-inflammatory effect of TG11–77.HCl on their cognitive abilities.

## Methods

### Ethics Statement

All experimental procedures involving animals were approved by the Emory University School of Medicine’s Animal Care and Use Committee and justified to the NIH guidelines for animal research.

### Animals

5xFAD-B6SJL transgenic mice (MMRRC Stock No: 34840-JAX) were used in the study.^8^ Colonies of 5XFAD mice are maintained by mating hemizygous transgenic male mice with female wild-type C57BL/6 x SJL F1 mice (B6/SJL F1 hybrid, Stock No: 100012, JAX). Both 5xFAD and nTg mice were housed together in auto water cages under 12 h light/dark cycle with ad libitum access to food and water. Mice were transferred manually to water when they were introduced to the drug treatment. There were two cohorts of mice used in the experimental paradigm. Cohort 1 mice were used as a single-hit (genetic) model from 8 to 20 weeks of their age. Cohort 2 mice were used as a two-hit (genetic and environmental) model and injected weekly once LPS (L2880, Sigma, USA) from 8 to 20 weeks of age (Figure 1). Totally, 141 mice were included (both sexes, 5xFAD and nTg) in the study. All mice were measured for their body weight, water consumption, and modified Irwin scores^29^ once every week.

### LPS and EP2 Antagonist Administrations

LPS (L2880, Sigma, USA) in sterile saline was injected intraperitoneally (IP) at 1.0 mg/kg of body weight once weekly from 8 to 20 weeks for all of the mice in cohort 2. TG11–77.HCl,^30^ the EP2 antagonist used in the study, is prepared freshly every week by dissolving in rodent drinking water at 0.5 mg/mL. For optimum solubility of the drug, the solution was adjusted to pH 3.5 by using diluted HCl. Similarly, control drinking water adjusted to pH 3.5 was used as the vehicle. The drug or vehicle solution was given to mice ad libitum in graduated glass drinking water bottles (Ancare, USA), and the volume was measured once weekly to calculate consumption. Based on the weekly measures of body weight, volume drunk, and at a measured 92.5% recovery of the drug from drinking water after 7 days at room temperature, the average rate of drug consumption was measured at 53 mg/kg/day in cohort 1 (single-hit) and 64.8 mg/kg/day in cohort 2 (two-hit). The rate of drug consumption in both cohorts is shown in Table S1 (Supporting Information).

### Tissue Collection and Processing

Mice were terminally perfused using ice-cold PBS 6h after the last LPS injection in cohort 2. Before perfusion, blood samples are collected by cardiac puncture and transferred to 4 °C until analyzed. Brains are harvested after perfusion, equally divided, longitudinally bisected, and appropriately stored for further processing. The cortex and hippocampus from one hemisphere were dissected and quickly transferred to dry ice and then subsequently to −80 °C for gene expression analysis. The other hemisphere was fixed overnight in 4% paraformaldehyde in 1× PBS (Sigma), sunk in 30% sucrose, and then stored in 4 °C until processed for immunohistochemical analysis.

### CBC Analysis

Freshly collected blood, by cardiac puncture using 1 mL syringes with 21G needles, is transferred to EDTA tubes and stored at 4 °C until sent for CBC analysis. CBC analysis was performed within 24 h of collection by the Quality Assurance & Diagnostic Lab, Division of Animal Resources, Emory University, using VetScan HM5 v2.3 Hematology Analyzer. 16 different blood cell parameters were analyzed, including the differential white blood cells (WBC), RBC, and platelet counts, PDWc for different cell types, and levels of HGB and HCT.

### qRT-PCR

Frozen cortices were homogenized using a sonicator in 1 mL of RNA extraction buffer supplied with the Quick-RNA MiniPrep Kit (Zymo Research). Homogenized tissue samples were centrifuged at 2000 rpm for 1 min to settle the tissue debris. 700 μL of the homogenate was used for RNA extraction as per the manufacturer’s suggestion in the protocol provided with the MiniPrep Kit. RNA samples were quantified using an Epoch Microplate Spectrophotometer (BioTek) and further converted to cDNA using a qScript cDNA SuperMix (Quanta Biosciences). Quantitative real-time polymerase chain reaction (qRT-PCR) is performed in a CFX96 Touch Real-Time PCR Detection System (Bio-Rad) using an iQ SYBR Green Supermix (Quanta Biosciences) with each sample run in technical duplicates. Three housekeeping genes, β-actin, glyceraldehyde-3-phosphate (GAPDH), and hypoxanthine phosphoribosyl transferase 1 (HPRT1), were used as internal controls. mRNA expression for cyclooxygenase-2 (COX-2), NADPH oxidase-2 subunits (gp91^phox^, p47^phox^), inducible nitric oxide synthase (iNOS), triggering receptor expressed on myeloid cells 2 (TREM2), interleukin 6 (IL-6), C–C motif chemokine ligands-2,-3,-4 (CCL2, CCL3, CCL4), tumor necrosis factor alpha (TNFα), interleukin 1-beta (IL-1β), and prostaglandin-E2 receptor (EP2), and genes related to astroglial and microglial markers, ionized calcium-binding adaptor molecule (Iba1), glial fibrillary acidic protein (GFAP), markers for infiltrating macrophages (CD11b, CD68), and S100 calcium-binding protein B (S100B), were measured. For qRT-PCR analysis, cycle threshold (CT) values for each gene of interest were normalized to their respective geometric means of CT values from 3 housekeeping genes. The fold changes in one group were measured using the 2^–ΔΔCt^ method by calculating the relative expression from their respective control groups.^31^ The fold changes were used for statistical analysis between groups. The primer sequences for different genes used for qRT-PCR are given in Table 1.

**Table 1:** Mouse Primer Sequences Used in qRT-PCR Reactions

genes	forward primer (sequence 5′–3′)	reverse primer (sequence 5′–3′)
βActin	AAGGCCAACCGTGAAAAGAT	GTGGTACGACCAGAGGCATAC
GAPDH	TGTCCGTCGTGGATCTGAC	CCTGCTTCACCACCTTCTTG
HPRT1	GGAGCGGTAGCACCTCCT	CTGGTTCATCATCGCTAATCAC
COX-2	CTCCACCGCCACCACTAC	TGGATTGGAACAGCAAGGAT
iNOS	CCTGGAGACCCACACACTG	CCATGATGGTCACATTCTGC
gp91^phox^	TGCCACCAGTCTGAAACTCA	TTGTCTAATGGGAACGTCACAC
p47^phox^	GCTGGTGGGTCATCAGGAAA	GCCCTGACTTTTGCAGGTACA
TREM2	GGTGCCATGGGACCTCTCCACCAGTTT	CTTCAGAGTGATGGTGACGGTTCCAGC
IL-6	TCTAATTCATATCTTCAACCAAGAGG	TGGTCCTTAGCCACTCCTTC
CCL2	CATCCACGTGTTGGCTCA	GCTGCTGGTGATCCTCTTGTA
CCL3	TGCCCTTGCTGTTCTTCTCT	GTGGAATCTTCCGGCTGTAG
CCL4	CATGAAGCTCTGCGTGTCTG	GGAGGGTCAGAGCCCATT
TNFα	TCTTCTGTCTACTGAACTTCGG	AAGATGATCTGAGTGTGAGGG
IL-1β	TGAGCACCTTCTTTTCCTTCA	TTGTCTAATGGGAACGTCACAC
EP2	TCTTTAGTCTGGCCACGATGCTCA	GCAGGGAACAGAAGAGCAAGGAGG
Iba1	GGATTTGCAGGGAGGAAAAG	TGGGATCATCGAGGAATTG
GFAP	GACAACTTTGCACAGGACCTC	ATACGCAGCCAGGTTGTTCT
CD11b	CCAGTAAGGTCATACAGCATCAGT	TTGATCTGAACAGGGATCCAG
CD68	CTC TCTAAGGCTACAGGCTGC T	TCA CGGTTGCAAGAGAAACA
S100B	TCGGACACTGAAGCCAGAG	AGACATCAATGAGGGCAACC

### Immunohistochemistry

Brain hemispheres were fixed in 4% paraformaldehyde (Sigma), transferred to 30% sucrose (Sigma) for 48 h, and then moved to PBS (Sigma) before being sent to the Neuropathology/Histochemistry Core of the Emory NINDS Neurosciences Core Facility for tissue section. As previously described,^32^ 7–10 brain samples from each group were processed as per the facility protocol and each alternate 10 μm paraffin-embedded coronal section was collected along the entire length of the hippocampus using a microtome (Leica).^32^ For immunofluorescent staining, an Iba1 (Wako, 019–19741, 1:500 dilution) primary antibody was used. The secondary antibody was Alexa Fluor 488 goat antirabbit (Thermofisher, A11008, 1:1000 dilution). Sections mounted on glass slides were deparaffined in xylene for 2 × 10 min and then hydrated in gradually decreasing concentrations of ethanol (100, 95, 75, and 50% in water). Slides were boiled in an antigen retrieval solution (DAKO) at 98 °C for 20 min and then slowly cooled to RT for 15 min. The sections were incubated in a solution containing 5% goat serum and 0.3% Triton-X in PBS for at least 1 h and then incubated with primary antibody (diluted in 2% goat serum and 0.3% Triton-X in PBS) overnight at 4 °C. Then, the sections were washed with PBS (3 × for 5 min each) and subsequently incubated for 2 h at room temperature in secondary antibody (diluted in 1% goat serum and 0.3% Triton-X in PBS). The sections were again washed with PBS. Finally, the sections were stained for Congo red (see below) or mounted with DAPI mounting media (Vectra Shield) for nuclear staining. The fluorescent images were captured with an AxioObserver A1 fluorescence microscope (Zeiss) using AxioVision AC 4.7 software (Zeiss). The same illumination intensity and image acquisition parameters were used to capture all of the images across different treatments. We restricted the staining to female treatment groups only as qRT-PCR analysis showed the effect of the drug only in female brains. 7–12 mice from each group and for each mouse 4 equidistance sections (every 20th section) from one hemisphere were selected for staining. Specific anatomical markers were used to ensure that the images were obtained from the same regions of the sections to reduce the variability. We intended to focus on different regions of brain hemi cortex, thalamus, and hippocampus for immunohistochemical quantification. Hence, we captured the brain sections under 20× magnified objectives. We also analyzed the data obtained from different regions to show any effect of the TG11–77.HCl treatment on immunoreactivity and the overall size and number of the amyloid plaques.

### Congo Red Staining

Four equidistant sections (every 20th section) from one hemisphere were selected for staining and measured for an average plaque count per mouse (comprising average numbers from 4 sections in each mouse) with 12–16 brain samples from each group. As described previously,^19^ after secondary antibody staining, brain sections were immediately transferred to 0.2% Congo red (Sigma) in saturated ethanolic NaCl solution.^19^ 1 mL of activator (1% NaOH) was freshly added to 100 mL of prestaining solution A (2.5% NaCl solution in 80% ethanol) or solution B (0.2% Congo red in 80% ethanolic NaCl solution) before use. Sections were incubated in solution A for 20 min and then in solution B for 30 min at room temperature. Finally, the slides were washed with PBS for 10 min and quickly destained using 80, 70, and 50% ethanol for 1 min each. After a final wash with dH_2_O, the slides were mounted with DAPI mounting media (Vectra Shield) for nuclear staining. Congo-red-positive plaques appear bright red when using a Rhodamine filter on the fluorescent microscope. The images were captured on an AxioObserver A1 fluorescence microscope (Zeiss) using AxioVision AC 4.7 software (Zeiss). The same illumination intensity and image acquisition parameters were used to capture all of the images across different treatments.

### Image Quantification

Using ImageJ software, green fluorescent images from Iba1-positive sections and red fluorescent images from Congo-red-stained sections were merged. Total numbers of particles, their size, and total area covered by fluorescence in each section were quantified. Every TIFF image was converted to (black and while) an 8-bit binary image and subjected to auto thresholding using one of the 16 built threshold filters in ImageJ. The same filter was used throughout. Using the “Analyze particles” feature, the number, size, and area were measured and further analyzed for graphical representation.

### Y-Maze Behavioral Assay

A Y-maze^33^ apparatus with three symmetrical arms, each 36 cm in length, was used. The ambient light of the testing room was dimmed to 20–25 lx to minimize anxiety. The rate of spontaneous alternation in arm choice was tested in a single-trial 3-arm Y-maze, in which each mouse was allowed to explore freely for 8 min. As shown by the composite heat maps in Figure S3, once a mouse committed to an arm, it tended to travel all the way to the end, so there was rarely any question whether a mouse had entered an arm. The number of choices resulting in an alternation (e.g., arm entry sequence of ACB but not ACA) was expressed as a fraction of the total number of choices. The number of arm entries in different mice ranged between about 20 and over 70 during the 8 min period.

### MWM Behavioral Assay

The MWM^34^ consists of a blue circular tank with a diameter of 120 cm and a height of 80 cm that is placed in the center of the room with visual cues on the walls (or on the maze wall) and filled with water (Temp. 23 °C). A hidden black platform, 12 cm in diameter, was located in the water (2 cm below the water surface), and the tank was conceptually divided into four quadrants with four points designed as starting positions (N, S, W or E). The mice performed four trials per day for 5 consecutive days. In the swimming trials, each individual mouse was placed in the water at a randomly chosen quadrant. During each trial, each mouse was given 60 s to find the hidden platform. If a mouse found the platform, it was allowed to stay on the platform for 15 s and then returned to the home cage. If the mouse could not find the platform within 60 s, the mouse was placed on the platform by hand for 15 s, and its escape latency was accepted as 60 s. The platform was camouflaged by placing opacifying materials in the water (typically, tempera paint) to create a nearly invisible platform-to-background color match. The interval between trials was 15–20 min. The time to reach the platform (latency), the length of swim path (distance), and the swim speed were measured. On the sixth day, subjects were tested on a probe trial, during which the escape platform was removed, and the time spent in the correct quadrant was measured for a 60 s trial.

### Statistical Analysis

Data were analyzed in GraphPad Prism 9. For body weight, two-way repeated-measure ANOVA with Sidak’s multiple comparisons test was applied. For CBC analysis, the unpaired *t*-test with the Bonferroni correction was applied between groups. For LPS effect on mRNA, we used one-way ANOVA with Dunnett’s multiple comparisons test. The paired *t*-test was applied between groups, in a series of proinflammatory and astroglial–microglial markers for mRNA analysis and in different regions of brains for immunohistochemical analysis. Data are presented as mean ± SEM for each group. P-values were set to be significant at * < 0.05, ** < 0.01 and *** < 0.001.

## Supplementary Material



## Notes

Measured consumption rate of TG11–77.HCl in mice (Table S1); impact of sex, transgene, and TG11–77,HCl treatment on body weight over the dosing period (Figure S1); CBC analysis of 5xFAD mice showing no impact of TG11–77.HCl treatment on anemia of inflammation in two-hit 5xFAD (Figure S2); and performance of 5xFAD mice on Y-maze with a vehicle or TG11–77.HCl treatment (Figure S3). This material is available free of charge on the ACS publications Web site (PDF)

## #

Department of Pharmaceutical Sciences, College of Pharmacy, University of Kentucky, Lexington, Kentucky 40506, United States

## ≠

Center for Innovative Drug Discovery, The University of Texas, San Antonio, Texas 78249, United States.
