# Supplementary material for: Antagonism of the EP2 Receptor Reveals Sex-Specific Protection in a Two-Hit Mouse Model of Alzheimer’s Disease
Source: ACS Chem Neurosci. 2026 Jan 2;17(2):392–403. doi: 10.1021/acschemneuro.5c00780 (PMC12828713; doi:10.1021/acschemneuro.5c00780)
Supplement: Supplementary file 1 [file cn5c00780_si_001.pdf]

Antagonism of the EP2 receptor reveals sex specific protection in a two-hit mouse model of Alzheimer's disease

Avijit Banik[#], Radhika Amaradhi[≠], Michael Sau, Varun Rawat, Raymond Dingledine, Thota Ganesh*

Department of Pharmacology and Chemical Biology, Emory University School of Medicine, Atlanta, GA 30322, US.

*Corresponding author: tganesh@emory.edu

Supporting Information:

Supporting Information (**SI**) below contains: **Table S1** describing the measured consumption rate of TG11-77.HCl in mice based on the consumed drug, impact of sex, transgene, and TG11-77,HCl treatment on body weight over the dosing period (**Fig S1**) indicating no impact of the drug on overall health, complete blood count (CBC) analysis of 5xFAD mice showing no impact of TG11-77.HCl treatment on anemia of inflammation in two-hit 5xFAD (**Fig S2**), and performance of 5xFAD mice on Y-maze with a vehicle or TG11-77.HCl treatment (**Fig S3**).

Table S1: Measured consumption rate of TG11-77.HCl in mice [a]

Time points	Body weight (g)	Solution drunk (ml/day)	Drug consumption (mg/kg/day)
Week 8	22.85	--	--
Week 9	22.78	3.00	65.81
Week 10	23.14	3.41	73.73
Week 11	24.24	3.50	72.26
Week 12	24.71	3.44	69.68
Week 13	25.00	3.41	68.23
Week 14	25.49	3.28	64.24
Week 15	25.77	3.41	66.07
Week 16	25.93	3.41	65.70
Week 17	26.08	3.32	63.74
Week 18	26.41	3.37	63.78

Week 19	26.55	3.35	63.01
Week 20	27.22	4.00	73.41
Average drug consumption			67.47 (mg/kg/day)
At 92.5% recovery			62.4 (mg/kg/day)
**Free base of TG11-77.HCl**			**53 (mg/kg/day)**

[a] The consumption rate was measured based on the weekly average body weight (g) and volume of drinking water consumed each day (ml). The drug was dissolved at the concentration of 0.5 mg/ml in drinking water. The final drug concentration was measured at 92.5% recovery of the actual dose from the drinking water with drug after 7 days of storage at room temperature. Formula used for daily drug consumption (mg/kg/day) = (solution consumed*0.5*1000)/body weight. Finally, the free base of the drug was estimated by multiplying the concentration with free base conversion factor (0.847).

Supporting Figures

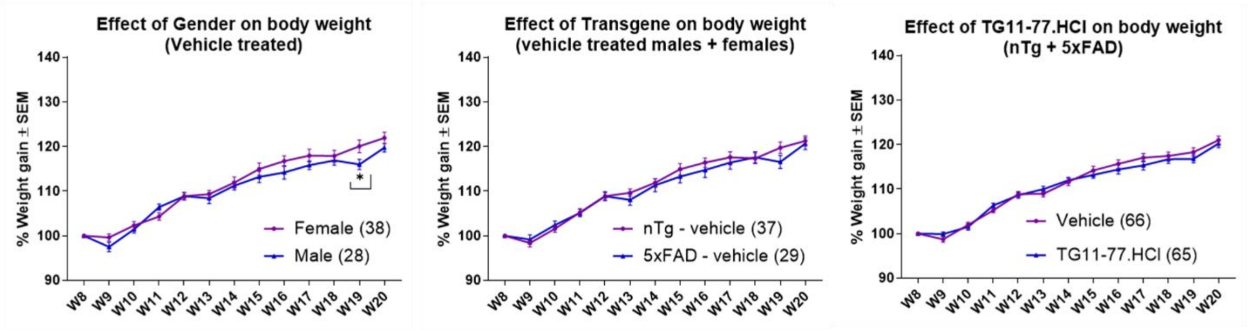

Fig. S1: There was no overall adverse effect of gender, transgene, and TG11-77.HCl treatment on body weight gain in different cohorts of mice. The % body weight gain was measured weekly once from week 8 to week 20. (A) % weight gain among vehicle treated females and males. (B) % weight gain among nTg and 5xFAD mice. (C) % weight gain upon TG11-77.HCl treatment in both nTg and 5xFAD mice. Two-way repeated measure ANOVA with Sidak's multiple comparisons test was applied. No statistical significance between groups was found on these A–C measures. Data are mean ± SEM.

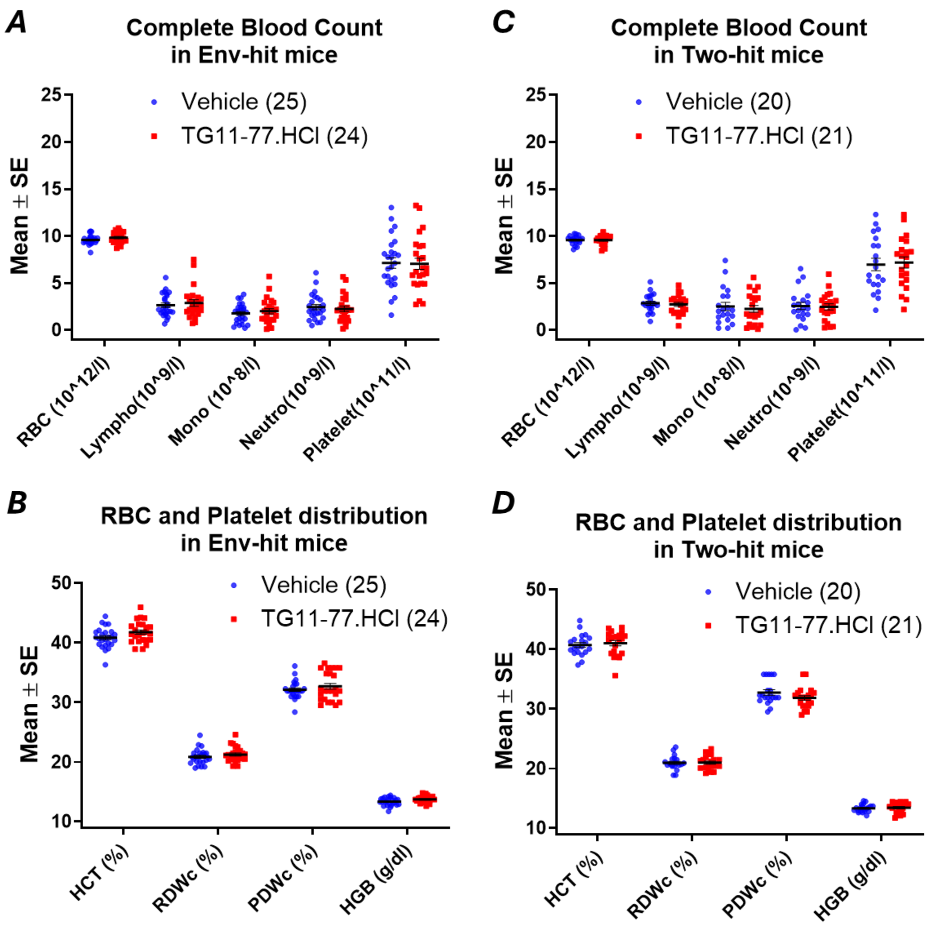

Fig. S2: Effect of TG11-77.HCl on the LPS induced anemia of inflammation. Both male and female mice (5xFAD and nTg) were injected with 1 mg/Kg LPS (intraperitoneal, weekly once) and treated with TG11-77.HCl or vehicle in drinking water from 8 to 20 weeks of their age. (A) In Env-hit cohort, TG11-77.HCl treatment did not alter the complete blood counts in terms of numbers of RBCs, lymphocytes, monocytes, neutrophils and platelets. (B) Similarly, there was no effect reported in the levels of hemoglobin (HGB), % hematocrit (HCT), RBC and platelet distribution in these mice after TG11-77.HCl treatment. In Two-hit mice also there was no effect of TG11-77.HCl treatment (C) on complete blood counts, and (D) RBC and platelet distribution. Multiple unpaired t test with Bonferroni correction was applied between groups. P values were set to be significant at * ≤ 0.05, ** ≤ 0.01 and *** ≤ 0.001. Data are mean ± SEM.

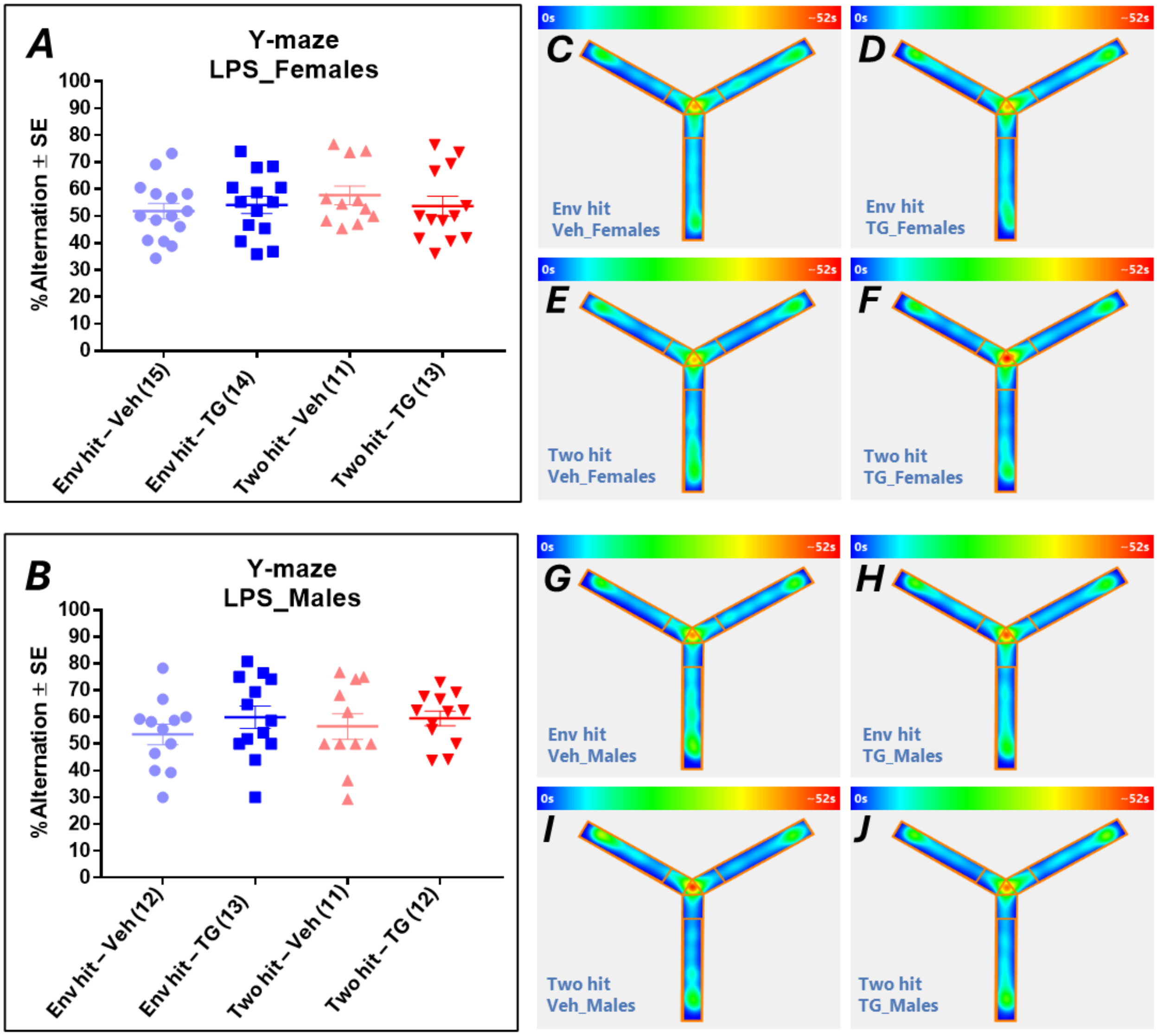

Fig. S3: Working memory performance in Y-maze by TG11-77.HCl or vehicle treated mice. Single trial of 8 minutes free exploration for each mouse was recorded to measure percent alternation in arm preference. (A) % alternation in Two-hit or Env-hit females treated either with vehicle of drug. (B) % alternation in Two-hit or Env-hit males treated either with vehicle of drug. (C-J) Heat maps of Y-maze trials from different groups showing exploratory behavior of these mice in three different arms of the Y-maze apparatus. Time spent inside the apparatus is represented by sea green→green→yellow with increasing time spent in each arm. One way ANOVA with Tukey's multiple comparison test was applied. Data are mean ± SEM.
